# Disparity of serum uric acid threshold for CKD among hypertensive and non-hypertensive individuals

**DOI:** 10.1080/0886022X.2023.2301041

**Published:** 2024-02-29

**Authors:** Bowen Zhu, Fang Li, Weidong Zhang, Shuan Zhao, Nana Song, Shi Jin, Ziyan Shen, Yufei Lu, Yang Li, Hong Liu

**Affiliations:** a Department of Nephrology, Zhongshan Hospital, Fudan University, Shanghai, China; b Shanghai Medical Center of Kidney, Shanghai, China; c Shanghai Key Laboratory of Kidney and Blood Purification, Shanghai, China

**Keywords:** Hypertension, serum uric acid, chronic kidney disease, CHARLS

## Abstract

**Introduction:**

Hypertension and rising serum uric acid (sUA) played a pivotal role in the development of Chronic Kidney Disease (CKD). This study investigates the interactive effect of sUA and hypertension on CKD and identifies the optimal threshold of sUA among individuals with and without hypertension in the Chinese community population.

**Materials and methods:**

The study included 4180 individuals aged 45–85 years, derived from the China Health and Retirement Longitudinal Study (CHARLS) between 2011 and 2015. Additionally, a hospital-based study enrolled subjects in the Department of Nephrology at Zhongshan Hospital, China from January 1, 2019, to December 31, 2021. The interaction effect analysis were used to assess the impact of sUA and hypertension on CKD. We also compared the distribution of sUA and the CKD risk in community populations, distinguishing between those with and without hypertension. For the hospital-based population, kidney injury was marked by a KIM-1 positive area.

**Results:**

Our results indicate a higher prevalence of CKD in the community population with hypertension (10.2% vs. 3.9%, *p* < .001). A significant additive synergistic effects of the sUA and hypertension on the CKD risk were found. When the sUA level was < 4.55 mg/dL in the hypertensive population and < 5.58 mg/dL in the non-hypertensive population, the risk of CKD was comparable (*p* = .809). In the propensity score matched (PSM) population, the result remained roughly constant.

**Conclusion:**

Therefore, even moderate levels of sUA was associated with a higher risk of CKD in middle-aged hypertensive patients, who warrant stricter sUA control.

## Introduction

Chronic Kidney Disease (CKD) is a progressive condition affecting over 10% of the global population, which amounts to more than 800 million individuals. CKD has emerged as one of the leading causes of mortality [1]. Increasing evidence suggests that elevated serum uric acid (sUA) may contribute to the development of CKD, rather than just being a marker of decreased renal uric acid excretion [2,3]. There is a documented correlation between sUA and hypertension, with a 2.21-fold increased risk of CKD [4–6]. Hypothesized mechanisms for uric acid-mediated renal damage include crystal deposition, oxidative stress, arteriolosclerosis, and glomerular hypertension [7]. The clustering of hypertension risk is elevated by sUA, and both conditions have been reported to contribute to kidney injury. However, the individual and combined roles of hypertension and sUA in CKD development are not fully understood [8–11].

Given the high prevalence of CKD in 31.8% of hypertensive patients and varying paradigms of hyperuricemia (HUA) in hypertension-related kidney injury, it is crucial to examine the association and distribution of sUA levels with CKD among hypertensive individuals. Studies have shown a relationship between sUA levels and CKD prevalence, indicating increased risk at specific sUA levels [12,13]. The underlying mechanisms between hypertension, sUA, and renal injury, are largely speculative. A proposed “2-hit” model suggests that urate plays a role in hypertension involving the activation of the renin-angiotensin system and inhibition of nitric oxide synthesis, followed by immune system involvement. These factors over time may increase the immune-inflammatory response in the kidney [7,14,15]. The varying associations and mechanisms between sUA and renal injury in individuals with and without hypertension suggest that the sUA threshold range for CKD might differ based on disease states.

A meta-analysis indicated that treating asymptomatic hyperuricemia could slow or delay CKD progression [16]. However, optimal sUA cutoff values of incident CKD among individuals with and without hypertension are yet not to be established. Guidelines from national and international rheumatology societies propose different sUA targets. The British Society for Rheumatology recommends a target below 5 mg/dL, while the European League Against Rheumatism and the American College of Rheumatology suggest a target below 6 mg/dL [17]. Early intervention for sUA less than 6.0 mg/dL is recommended for slowing CKD progression in Japan [18]. In China, the optimal sUA level target for reducing CKD risk in different disease states remains unclear. Our study aims to explore the interactive effect of sUA and hypertension on CKD risk and to identify the optimal sUA threshold for CKD among individuals with and without hypertension utilizing data from the China Health and Retirement Longitudinal Study(CHARLS) and Zhongshan Hospital, China.

## Materials and methods

### Subjects and study design

The CHARLS is an ongoing nationwide prospective cohort study comprised individuals over the age of 45 in China from 2008 to 2018, aimed at facilitating scientific research on the elderly. A multistage probability sampling method was designed to randomly select residential households from 450 villages and resident communities in 150 counties and districts located in 28 provinces. Details on the design and data collection of CHARLS are elaborated in prior publications [19]. Data encompassing demographic information, health status and functioning, and social and economic status were systematically collected from each participant throughout all waves of CHARLS. Written informed consent was obtained from each participants. The CHARLS was approved by the biomedical ethics committee of Peking University. The initial national wave was launched in 2011, encompassing approximately 10,000 households and 17,500 individuals spanning 150 counties/districts and 450 villages/urban communities. Subsequent follow-ups were conducted biennially, denoted as wave 2 and wave 3 in 2013 and 2015, respectively. This particular study was grounded in the cohort tracked from 2011 to 2015, where blood samples were obtained in the initial and final years. After integrating demographic, clinical examination, medication, and biomarker data, 8,356 participants were included from the 2011 wave. The cohort was narrowed down to 4,180 participants for the present analysis, excluding those outside the 45–75 age range, missing baseline serum creatinine (Scr), serum uric acid (sUA), systolic blood pressure (SBP) data (*n* = 218), lost to follow-up (*n* = 1,859), or missing follow-up Scr data (*n* = 1,568). The selection process is illustrated in Supplementary Figure 1.

For the hospital-based component, participants were recruited from the Department of Nephrology at Zhongshan Hospital, China, between January 1, 2019, and December 31, 2021. This study was sanctioned by the Zhongshan Hospital Institutional Review Board approved this retrospective study following the principles of the Declaration of Helsinki (B2021-740). Written informed consent was obtained from all the patients. Exclusion criteria included: ① Secondary hyperuricemia (such as that associated with myeloproliferative disorder); ② Severe renal impairment (estimated glomerular filtration rate [eGFR] under 15 mL/min/1.73m^2^); ③ Any medical condition that, per the investigator’s discretion, could impede treatment efficacy, patient safety, or protocol adherence. The screening assessment for subjects involved a physical examination, vital sign measurements, medical history, comprehensive lab testing (including a complete chemistry panel and hematology), current medication review, and examination of renal tissue pathology. Post-exclusion, 20 hypertensive individuals and 20 age-matched control patients, aged 18–75 years without prior renal impairment diagnoses (eGFR under 60 mL/min/l.73 m^2^ or presence of albuminuria), were deemed eligible (Supplementary Figure 2).

### Data collection

A standardized structured questionnaire was administered by trained health staff using a face-to-face computer-assisted personal interviews (CAPI) in 2011. The collected socio-demographic variables include age, gender, ethnicity, educational attainment, urban or rural residence, history of diseases (e.g. hypertension, diabetes), lifestyle and health-related behaviors (smoking, and drinking), health status and functionality. Physical examinations encompassing height, weight, waist circumference, blood pressure, walking speed, and balance tests were performed by trained clinical staff. Following household interviews, participants were invited to local health facilities or China CDC offices for blood sample collection by trained nurses. Complete blood counts (CBC) were conducted promptly after sample collection, and blood specimens were managed and preserved for subsequent analysis of hemoglobin A1C (HbA1C) and other biomarkers. These samples were eventually transported to Beijing and preserved at −70 °C at the China CDC. The biomarker profile from the CHARLS 2011 data release included 16 blood parameters, such as white blood cell count (WBC), mean corpuscular volume (MCV), platelets, blood urea nitrogen (BUN), blood glucose, creatinine, total cholesterol (TC), triglycerides (TG), low-density lipoprotein (LDL) cholesterol, C-reactive protein (CRP), glycosylated hemoglobin (HbA1c), serum uric acid (sUA), hematocrit (HCT), hemoglobin (Hb), and Cystatin C. Participants were categorized into groups based on ethnicity (Han and others), residential status (urban and rural), education level, smoking status, and drinking habits. Body mass index (BMI) was calculated as weight in kilograms divided by height in meters squared (kg/m^2^), with classifications into lean, normal, overweight, and obese based on established thresholds. Cutoff points for waist circumference were set in accordance with WHO guidelines [20]. Diabetes mellitus was identified if self-reported or based on diabetes mellitus treatment records or HbA1C levels [21]. Dyslipidemia was defined as a TC ≥5.2 mmol/L, LDL-C ≥3.4 mmol/L, or TG ≥1.7 mmol/L [22].

In the hospital-based study, the socio-demographic data captured included age, gender, medical history, smoking and drinking habits, and physical measurements such as weight, height, and BMI. Clinical biomarkers obtained from hospital records included ALT, AST, creatinine, urea, sUA, eGFR, urinary proteins, HbA1c, fasting glucose, and lipid profiles (HDL, LDL, TG, TC).

### SUA, HTN, and eGFR measurements

The eGFR was calculated using the Chronic Kidney Disease Epidemiology Collaboration formula (CKD-EPI) [23]. CKD was defined as an eGFR lower than 60 mL/min/1.73 m^2^. sUA levels were stratified into <4.55, 4.55–5.24, 5.25–6.35, >6.35 mg/dL for males and <3.28, 3.28–3.86, 3.87–4.57, >4.57 mg/dL for females in all population; and <4.06, 4.06–4.75, 4.76–5.68, >5.68 mg/dL for males and <3.70, 3.70–3.90, 3.91–5.72, >5.72 mg/dL for females mg/dL in PSM population. The systolic blood pressure (BP) and diastolic BP were expressed as the mean of three measurements. Hypertension was defined as systolic BP ≥ 140 mmHg or diastolic BP ≥ 90 mmHg or self-reported [24].

### Histologic examination and staining

Renal biopsy tissue was fixed in 4% paraformaldehyde for paraffin-embedded kidney sections (4 µm), which were then deparaffinized and rehydrated for the following staining techniques. For histologic examination, H&E staining was performed using the standard methods. Briefly, rehydrated kidney sections were subjected to antigen retrieval using the Antigen Unmasking Solution purchased from Vector, followed by blocking with 2% normal goat serum. The sections were then incubated with primary antibodies at the following dilutions: KIM-1(1:200) (MA5-43897, Thermo Fisher Scientific, US), CD4 (1:100) (ab231460, Abcam, US), CD68 (1:100) (ab955, Abcam, US) overnight at 4 °C, washed three times in PBS, incubated with appropriate secondary antibodies, and washed with PBS again. For immunohistochemistry, the signals were visualized using VECTASTAIN ABC kits (Vector), followed by counterstaining with hematoxylin and capturing images using a CX31 microscope with a DP73–1-51 digital camera (Olympus). For immunofluorescence staining, after incubation with the primary antibodies indicated and washing with PBS, the sections were incubated with DyLight 488-conjugated secondary antibodies at 37 °C for 1 h. Nuclei were counterstained with DAPI (1:1000; Sigma-Aldrich). Images were captured by the Vectra Imaging System. KIM-1 positive area in glomeruli was measured by the Image J software.

### Statistical analysis

A propensity score-matched (PSM) cohort was constructed to control for hypertension, with matching based on age, gender, educational levels, presence of diabetes mellitus, smoking habits, and alcohol consumption. Continuous variables were described as means with standard deviations (SD), while categorical variables were represented as counts and percentages (N (%)). Demographic and clinical characteristics were compared between groups using the T-test, *χ*^2^ test, or the Fisher exact test where appropriate. Furthermore, we examined the interaction effect of the sUA quartiles and hypertension (or elevated systolic pressure/elevated diastolic pressure) on the incident CKD. The univariate, fully adjusted multivariate, and bidirectional stepwise logistic regression model with an entry and exit level of significance of 0.1 was used to identify independent predictors of CKD. To quantify the synergistic effect, indicators of relative excess risk due to interaction (RERI), attributable proportion (AP) due to interaction, and synergy index (SI) were calculated. When the 95% confidence interval (CI) for RERI, AP, and SI did not overlap 0, 0, and 1, respectively, a significant additive interaction was observed. We further conducted the stratified analysis classified by hypertension (systolic BP or diastolic BP) to investigate associations of the sUA quartiles with CKD among individuals. Multivariate logistic regression models were adjusted in sequence for age (treated as a continuous variable), gender, BMI, waist-to-hip ratio (WHR), diabetes mellitus, smoking status, and alcohol consumption. Additionally, we executed a dose-response analysis to assess the relationship between incremental sUA levels and CKD risk, incrementing the sUA cutoff from 3 to 8 mg/dL by 0.1 mg/dL increments within the CHARLS dataset. Outcomes were presented as risk ratios (RR) with corresponding 95% confidence intervals (CI). A two-sided *p* of less than .05 (two-sided) was regarded as statistically significant. All analyses were conducted on the dataset available, using SAS software version 9.3 (SAS Institute, Cary, NC). To further validate our findings, multiple sensitivity analyses were performed, examining the additive interaction effects of sUA quartiles with systolic and diastolic blood pressure on CKD. The associations of sUA quartiles with CKD were also analyzed within subgroups defined by systolic and diastolic blood pressure levels.

## Result

### Characteristics of the study participants

Over the 5-year follow-up in CHARLS, the incidence of Chronic Kidney Disease (CKD) was 10.2% (167/1639) among hypertensive individuals and 3.9% (98/2541) among non-hypertensive individuals. Hypertensive participants were older on average and more likely to have a non-Han nationality, junior-school education, a history of cardiovascular disease (CVD), overweight, dyslipidemia, elevated levels of serum uric acid (sUA), and usage of antihyperglycemic and lipid-lowering drugs. They also had lower rates of smoking and drinking. Following propensity score matching (PSM), 1354 hypertensive individuals were matched with an equal number of non-hypertensive individuals based on age, gender, education, BMI, diabetes mellitus status, and tobacco and alcohol use. The CKD incidence rates post-matching were 7.9% (107/1354) for the hypertensive group and 5.1% (69/1354) for the non-hypertensive group (Table 1).

**Table 1.** Baseline characteristics of population with and without hypertension.

	Hypertension (*n* = 1639)	Non-Hypertension (*n* = 2541)	*p-value*	Propensity Hypertension (*n* = 1354)	Matched Non-Hypertension (*n* = 1354)	*p-value*
Demographics						
Age, years	61.8 (8.5)	58.4 (8.2)	<.001	60.8 (8.2)	60.9 (8.4)	.803
Male	724 (44.2)	1191 (46.9)	.087	609 (44.9)	630 (46.4)	.418
Married/cohabitating	1236 (75.4)	2021 (79.5)	<.001	1024 (75.5)	1052 (77.5)	.205
Han Nationality	1499 (91.5)	2380 (93.7)	.007	1238 (91.2)	1276 (94.0)	.005
Rural	1374 (83.9)	2173 (85.6)	.133	1159 (85.4)	1149 (84.7)	.537
Education, years			.013			.852
0	504 (30.8)	688 (27.1)		403 (29.7)	419 (30.9)	
1–6	748 (45.6)	1161 (45.7)		629 (46.4)	608 (44.8)	
7–9	267 (16.3)	456 (18.0)		226 (16.7)	226 (16.7)	
>9	120 (7.3)	236 (9.3)		99 (7.3)	104 (7.7)	
History of CVD	324 (19.8)	211 (8.3)	<.001	257 (18.9)	133 (9.8)	<.001
Current smoker			.040			.581
Never	1009 (61.6)	1544 (60.9)		823 (60.7)	825 (60.8)	
Ever	147 (9.0)	181 (7.1)		114 (8.4)	100 (7.4)	
Current	481 (29.4)	809 (31.9)		420 (31.0)	432 (31.8)	
Current drinker			<.001			.706
Never	1156 (70.5)	1639 (64.5)		931 (68.6)	912 (67.2)	
Ever	117 (7.1)	225 (8.9)		108 (8.0)	109 (8.0)	
Current	366 (22.3)	676 (26.6)		318 (23.4)	336 (24.8)	
Physical Examination						
BMI (kg/m^2^)			<.001			.891
Lean (<18.5)	57 (3.5)	186 (7.4)		56 (4.1)	61 (4.5)	
Normal (18.5–23.9)	735 (45.4)	1509 (60.1)		688 (50.7)	695 (51.2)	
Overweight (24–27.9)	540 (33.4)	635 (25.3)		445 (32.8)	444 (32.7)	
Obesity (≥28.0)	286 (17.7)	182 (7.3)		168 (12.4)	157 (11.6)	
Waist circumference, cm	1117 (68.4)	1193 (47.2)	<.001	860 (63.4)	801 (59.0)	.013
≥90 for male or 85 for female						
Comorbidities						
Systolic BP, mm Hg	147 (20.5)	118.2 (11.7)	<.001	146.6 (20.3)	119.3 (11.7)	<.001
Diastolic BP, mm Hg	83.4 (12.1)	70.3 (9.0)	<.001	83.6 (12.1)	70.3 (8.8)	<.001
Diabetes mellitus	188 (11.5)	135 (5.3)	<.001	111 (8.2)	101 (7.4)	.474
Dyslipidemia	686 (41.9)	876 (34.5)	<.001	558 (41.1)	494 (36.4)	.012
sUA, mg/dL			<.001			<.001
Q1 (males: <4.06; females: <3.28)	338 (20.6)	707 (27.8)		322 (23.7)	355 (26.2)	
Q2 (males: 4.06–4.75; females: 3.28–3.86)	376 (22.9)	668 (26.3)		327 (24.1)	352 (25.9)	
Q3 (males: 4.76–5.68; females: 3.87–4.57)	412 (25.1)	634 (25.0)		335 (24.7)	344 (25.4)	
Q4 (males: >5.68; females: >4.57)	513 (31.3)	532 (20.9)		373 (27.5)	306 (22.6)	
Blood tests						
sUA (mg/dL)	4.6 (1.3)	4.3 (1.2)	<.001	4.5 (1.3)	4.4 (1.2)	.022
Total cholesterol, mmol/L	197.5 (39.4)	190 (38.1)	<0.001	196.7 (39.6)	193 (39.5)	.015
HDL cholesterol, mmol/L	49.1 (15.0)	52.2 (15.4)	<.001	50 (15.1)	50.4 (14.4)	.408
LDL cholesterol, mmol/L	117.3 (36.2)	113.4 (33.4)	<.001	116.5 (35.7)	116.3 (34.4)	.854
Triglycerides (mg/dL)	153.3 (125.9)	123.9 (98.4)	<.001	148.2 (119.8)	131.1 (101.8)	<.001
HbA1c, %	5.3 (0.9)	5.2 (0.7)	<.001	5.3 (0.8)	5.3 (0.8)	.808
Fasting Glucose	113.3 (41.8)	105.8 (28.8)	<.001	111 (38.0)	107.6 (29.3)	.01
Creatine, mg/dL	0.84 (0.20)	0.81 (0.20)	<.001	0.79 (0.20)	0.77 (0.17)	.02
Medication Use						
Antihyperglycemic drugs	88 (5.4)	45 (1.8)	<.001	48 (3.5)	35 (2.6)	.174
Antihypertensive drugs	808 (49.3)	0 (0.0)		638 (47.0)	0 (0.0)	
Lipid-lowering drugs	158 (9.6)	68 (2.7)	<.001	120 (8.8)	53 (3.9)	<.001

BMI: body mass index; BP: blood pressure; CKD: chronic kidney disease; CVD: cardiovascular disease; eGFR: estimated glomerular filtration rate; HDL: high-density lipoprotein; LDL: low-density lipoprotein; sUA: serum uric acid. a, sUA levels were categorized into <4.55, 4.55–5.24, 5.25–6.35, >6.35 mg/dL for males and <3.28, 3.28–3.86, 3.87–4.57, >4.57 mg/dL for females in all population; and <4.06, 4.06–4.75, 4.76–5.68, >5.68 mg/dL for males and <3.70, 3.70–3.90, 3.91–5.72, >5.72 mg/dL for females mg/dL; Data are presented as No. (%), mean ± SD or median (IQR); *p values were calculated by using T test or Wilcoxon test for continuous variables and *χ*^2^ test or fisher exact test for categorical variables.

### Predictors of Chronic Kidney Disease

Multivariate analysis revealed that age, hypertension, and sUA were independent predictors of CKD across the entire population. In the PSM cohort, additional factors such as marital status also emerged as predictors. Utilizing bidirectional logistic regression methods, older age, hypertension, and elevated sUA levels continued to be significant predictors of CKD (Table 2).

**Table 2.** Independent predictors of chronic kidney disease at 4-year follow-up in all and PSM population.

	All population			PSM population		
	Unadjusted RR (95% confidence interval)	Fully adjusted RR (95% confidence interval)	Bidirectional RR (95% confidence interval)	Unadjusted RR (95% confidence interval)	Fully adjusted RR (95% confidence interval)	Bidirectional RR (95% confidence interval)
Age, per 5 years increase	1.8 (1.7–2.0)***	1.8 (1.7–2.0)***	1.8 (1.6–1.9)***	1.8 (1.6–2.0)***	1.8 (1.6–2.0)***	1.8 (1.6–1.9)***
Male	1.0 (0.7–1.3)	1.1 (0.7–1.6)		0.9 (0.7–1.2)	1.0 (0.6–1.5)	
Married/cohabitating	0.6 (0.5–0.8)***	1.2 (0.8–1.6)		0.7 (0.5–0.9)*	1.3 (0.9–1.9)	
Rural	1.6 (1.1–2.1)**	1.2 (0.9–1.8)		1.6 (1.1–2.3)*	1.6 (1.0–2.4)*	
Education, years						
0	1.00 (ref)	1.00 (ref)		1.00 (ref)	1.00 (ref)	
1–6	0.8 (0.6–1.0)	1.1 (0.8–1.6)		0.9 (0.6–1.2)	1.2 (0.8–1.8)	
7–9	0.6 (0.4–0.9)**	1.4 (0.9–2.3)		0.6 (0.3–1.0)*	1.2 (0.7–2.3)	
>9	0.5 (0.3–0.9)*	0.8 (0.4–1.7)		0.4 (0.2–0.8)**	0.6 (0.2–1.7)	
History of CVD	1.7 (1.2–2.3)***	1.2 (0.8–1.7)		1.5 (1.0–2.2)*	1.3 (0.8–2.0)	
Current smoker						
Never	1.00 (ref)	1.00 (ref)		1.00 (ref)	1.00 (ref)	
Ever	1.2 (0.8–1.9)	1.0 (0.6–1.8)		1.0 (0.6–1.8)	0.9 (0.5–1.7)	
Current	0.9 (0.7–1.2)	1.0 (0.7–1.5)		1.0 (0.7–1.4)	1.0 (0.6–1.5)	
Current drinker						
Never	1.00 (ref)	1.00 (ref)		1.00 (ref)	1.00 (ref)	
Ever	0.7 (0.4–1.1)	0.9 (0.5–1.6)		0.9 (0.5–1.5)	1.0 (0.5–1.8)	
Current	0.8 (0.6–1.1)	0.8 (0.6–1.2)		0.8 (0.6–1.2)	0.7 (0.5–1.1)	
BMI (kg/m^2^)						
Lean (<18.5)	1.00 (ref)	1.00 (ref)		1.00 (ref)	1.00 (ref)	
Normal (18.5–23.9)	0.6 (0.4–1.0)*	0.8 (0.5–1.4)		0.4 (0.3–0.8)**	0.7 (0.4–1.3)	
Overweight (24–27.9)	0.7 (0.4–1.2)	0.9 (0.5–1.6)		0.4 (0.2–0.7)**	0.7 (0.3–1.6)	
Obesity (≥28.0)	1.0 (0.6–1.8)	1.3 (0.7–2.5)		0.4 (0.2–0.8)**	1.0 (0.4–2.3)	
WC, ≥90 for male or 85 for female, cm	1.3 (1.0–1.7)*	1.0 (0.7–1.5)		0.9 (0.7–1.2)	1.1 (0.7–1.7)	
Hypertension	2.8 (2.2–3.7)***	1.7 (1.3–2.3)***	1.8 (1.4–2.4)***	1.6 (1.2–2.2)**	1.6 (1.1–2.2)	1.6 (1.1–2.2)**
Diabetes mellitus	1.2 (0.8–1.9)	0.8 (0.5–1.4)		0.6 (0.3–1.2)	0.6 (0.3–1.3)	
Dyslipidemia	1.1 (0.8–1.4)	0.8 (0.6–1.1)		0.9 (0.7–1.3)	0.9 (0.6–1.2)	
sUA, mg/dL^a^						
Q1 (males: <4.06; females: <3.28)	1.00 (ref)	1.00 (ref)	1.00 (ref)	1.00 (ref)	1.00 (ref)	1.00 (ref)
Q2 (males: 4.06–4.75; females: 3.28–3.86)	2.4 (1.4–4.2)**	1.9 (1.1–3.4)*	1.9 (1.1–3.4)*	2.1 (1.1–4.1)*	2.0 (1.0–3.9)	1.5 (0.8–2.9)
Q3 (males: 4.76–5.68; females: 3.87–4.57)	3.7 (2.2–6.2)***	3.1 (1.8–5.4)***	3.2 (1.8–5.5)**	3.8 (2.0–7.1)**	3.7 (2.0–7.1)***	2.3 (1.3–4.2)**
Q4 (males: >5.68; females: >4.57)	9.0 (5.5–14.8)***	7.0 (4.2–11.7)***	7.1 (4.2–11.8)***	7.7 (4.3–13.9)**	7.4 (4.0–13.7)	5.5 (3.2–9.6)***
Medication Use						
Antihyperglycemic drugs	1.5 (0.8–2.7)			1.0 (0.5–1.8)		
Antihypertensive drugs	2.7 (2.1–3.5)***			1.8 (1.3–2.5)***		
Lipid-lowering drugs	1.3 (0.8–2.1)	0.8 (0.4–1.4)		1.2 (0.7–2.3)	0.8 (0.4–1.6)	

Abbreviation: BMI, body mass index; BP, blood pressure; CKD, chronic kidney disease; CVD: cardiovascular disease; eGFR, estimated glomerular filtration rate; sUA, serum uric acid; HDL, high-density lipoprotein; LDL, low-density lipoprotein; PSM, propensity score matching; RR, risk ratio; WC, waist circumference. Fully adjusted for all listed variables in Logistic regression models. a, SUA levels were categorized into <4.55, 4.55–5.24, 5.25–6.35, >6.35 mg/dL for males and <3.28, 3.28–3.86, 3.87–4.57, >4.57 mg/dL for females in all population; and <4.06, 4.06–4.75, 4.76–5.68, >5.68 mg/dL for males and <3.70, 3.70–3.90, 3.91–5.72, >5.72 mg/dL for females mg/dL in PSM population.).

*p < .05; **p < .01; ***p < .001.

### Interactive effect of sUA and hypertension on Chronic Kidney Disease

**Figure 1.**
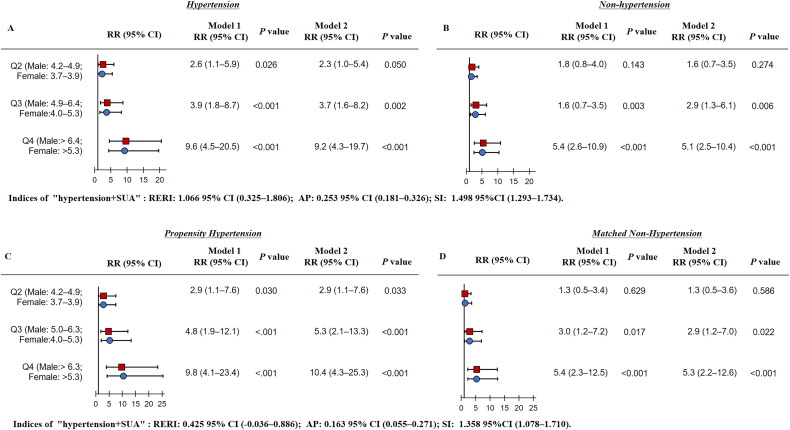
Logistic analyses of sUA quartiles and CKD in the hypertension and non-hypertension. (A) sUA quartiles and CKD risk among the hypertensive individuals; (B) sUA quartiles and CKD risk among the non-hypertensive individuals; (C) sUA quartiles and CKD risk among the propensity hypertensive individuals; (D) sUA quartiles and CKD risk among the matched non-hypertensive individuals).

Figure 1 illustrates the synergistic interaction between sUA levels and hypertension on CKD incidence. Calculations of relative excess risk due to interaction (RERI), attributable proportion (AP), and synergy index (SI) produced values of 1.066 (95% CI: 0.325–1.806), 0.253 (95% CI: 0.181–0.326), and 1.498 (95% CI: 1.293–1.734), respectively. These results indicate a significant additive interaction between sUA and hypertension in relation to CKD. Stratified analyses by hypertension status demonstrated a significant association between higher sUA quartiles and increased CKD risk, with a linear trend. After adjusting for continuous age, gender, BMI, waist circumference, diabetes, dyslipidemia, smoking, and alcohol intake, the multivariate-adjusted risk ratio (RR) of the highest sUA quartile (Q4) for CKD was 9.2 (95% CI: 4.3–19.7) in hypertensive individuals and 5.1 (95% CI: 2.5–10.4) in non-hypertensive individuals. Within the PSM cohort, the multivariate-adjusted RR of sUA Q4 for CKD was 7.1 (95% CI: 3.1–6.2) in hypertensive participants and 4.1 (95% CI: 1.9–9.1) in their non-hypertensive counterparts (Figure 1). These additive interactions were consistent across subgroups defined by systolic blood pressure (Supplementary Tables 1 and 2).

**Figure 2.**
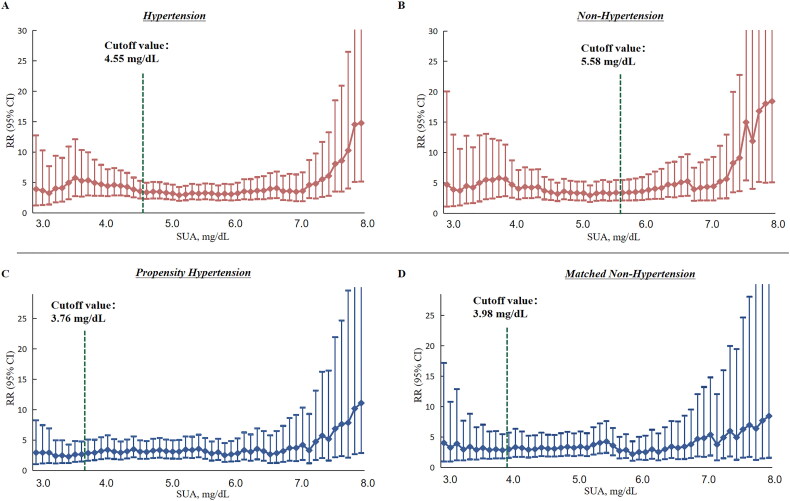
Multiple-adjusted risk ratios and 95% confidence intervals of CKD associated with sUA threshold ((A) risk for CKD by threshold sUA among the hypertensive individuals; (B) risk for CKD by threshold sUA among the non-hypertensive individuals; (C) risk for CKD by threshold sUA among the propensity hypertensive individuals; (D) risk for CKD by threshold sUA among the matched non-hypertensive individuals).

Figure 2 and Supplementary Table 3 display the stable CKD risk relative to sUA levels up to a turning point—4.55 mg/dL for hypertensive and 5.58 mg/dL for non-hypertensive populations. In the PSM cohort, these thresholds were 3.66 mg/dL and 3.98 mg/dL, respectively. CKD risk was comparable when sUA levels were below these points (*p* = .809 and *p* = .980, respectively) (Table 3).

**Table 3.** Multivariable-adjusted risk ratios of controlled targets for Chronic kidney Disease according to the presence of sUA and hypertension status.

	Incidence of CKD [n (%)]	Age- and sex-adjusted RR	P value	Fully-adjusted RR	P value
All population					
Non-hypertension and sUA < 5.58 mg/dL	69 (3.2)	1.00 (ref)		1.00 (ref)	
Hypertension and sUA < 4.55 mg/dL	44 (5.0)	1.1 (0.7–1.6)	0.691	1.1 (0.7–1.6)	0.809
Non-hypertension and sUA ≥ 5.58 mg/dL	29 (8.1)	2.6 (1.6–4.3)	<0.001	2.9 (1.8–4.6)	<0.001
Hypertension and sUA ≥ 4.55 mg/dL	123 (16.3)	4.4 (3.2–6.1)	<0.001	4.1 (2.9–5.8)	<0.001
PSM population					
Non-hypertension and sUA < 3.98 mg/dL	10 (2.3)	1.00 (ref)		1.00 (ref)	
Hypertension and sUA < 3.76 mg/dL	13 (2.6)	1.0 (0.4–2.4)	0.951	1.0 (0.4–2.4)	0.980
Non-hypertension and sUA ≥ 3.98 mg/dL	59 (6.4)	2.7 (1.4–5.5)	0.005	2.7 (1.3–5.4)	0.006
Hypertension and sUA ≥ 3.76 mg/dL	94 (11.1)	5.5 (2.8–11.0)	<0.001	5.4 (2.7–10.7)	<0.001

Abbreviation: sUA, serum uric acid; RR, risk ratio; Fully-adjusted were adjusted age (as continuous), gender, BMI, WHR, diabetes mellitus, smoking status, and alcohol intake.

### Evaluation mechanism of sUA on the Chronic Kidney Disease risk among hypertensive and non-hypertensive individuals

**Figure 3.**
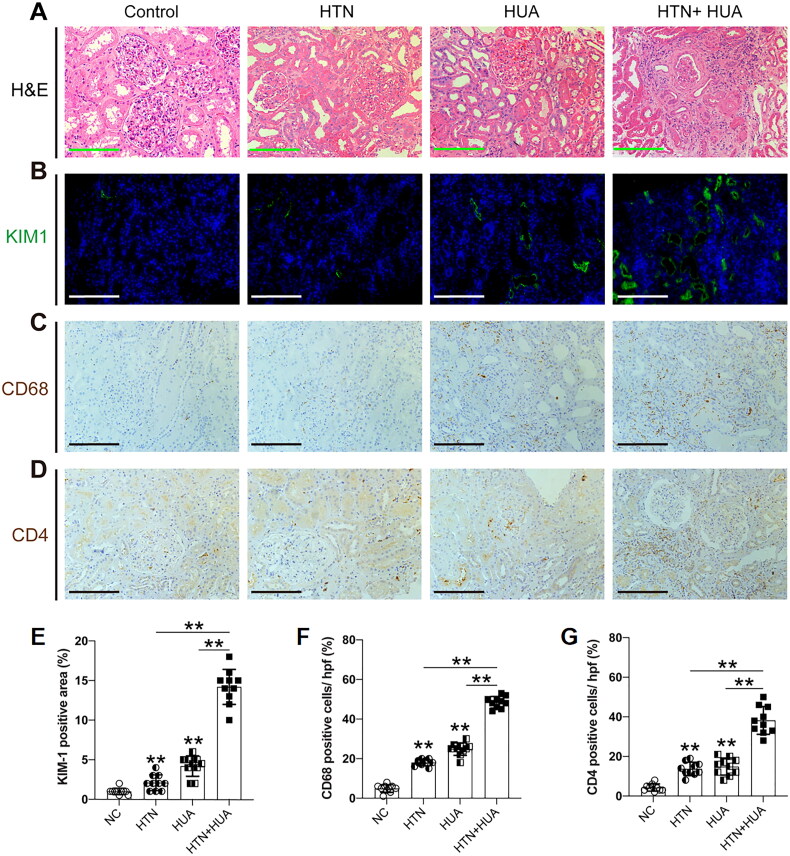
Hypertension and HUA jointly promote renal inflammation. ((A) representative micrographs show kidney morphology by H&E staining; (B,E) expression level of kidney injury marker KIM-1 is increased in hypertensive patient and hyperuricemia patients, compared to the control group, which was further increased in patients who developed both hypertension and hyperuricemia; (C,F) immunohistochemical staining revealed an increased infiltration of CD68+ macrophage; (D,G) CD4+ T cells in kidney of hyperuricemia patient and HTN patient, respectively, compared to the control group). And were further increased in patients who developed both HTN and HUA. 10 randomly selected fields of each kidney were counted, data are presented as mean ± SD from 10 patients per group, ** *p* < .010.

In the hospital-based study, a total of 40 patients were eligible. Among these, 20 hypertensive individuals and 20 non-hypertensive age-matched controls were enrolled in this study. The hypertensive group consisted predominantly of men (85.0%), as did the control group (75.0%). These groups were further divided into subgroups based on the presence of hyperuricemia. Among the hypertensive individuals, the mean eGFR in hyperuricemia subjects and non-hyperuricemia individuals was 34.2 ± 21.3 mL/min/l.73m^2^ and 49.5 ± 29.2 mL/min/l.73m^2^, respectively, and the difference was found to be not statistically significant (*p* < 0.001). Notably, higher urea levels and a greater prevalence of diabetes were found in hyperuricemic participants across both groups (*p* < .05) (Table 4). Kidney injury molecule-1 (KIM-1), a marker of renal tubular injury, was elevated in individuals with both hypertension and hyperuricemia, correlating with increased numbers of CD4+ T cells and macrophages (CD68+). These findings are further detailed in Figure 3.

### Sensitivity and explanatory analyses

Subgroup analyses based on systolic and diastolic blood pressure levels revealed persistent additive interactions between systolic blood pressure and sUA on CKD risk. The associations between sUA quartiles and CKD remained consistent when stratified by blood pressure categories (Supplementary Figures 1 and 2).

**Table 4.** Baseline characteristics of the individuals stratified by hypertension and hyperuricemia in the Zhongshan Hospital.

	HTN		*P* value	Non-HTN		*P* value
	Hyperuricemia	Non-Hyperuricemia		Hyperuricemia	Non-Hyperuricemia	
Participants (*n*)	10	10		10	10	
*Demographics*						
Age, years	53.9 ± 7.6	49.1 ± 14.9	0.213	40.7 ± 15.3	35.4 ± 19.1	0.501
Male	9 (88.9)	8 (90.0)	0.937	9 (90.0)	6 (60.0)	0.302
Married status	10 (100.0)	8 (80.0)	0.279	7 (70.0)	5 (50.0)	0.361
*Anthropometry parameters*						
Weight, kg	73.3 ± 4.5	81.9 ± 13.0	0.108	71.6 ± 17.9	68.0 ± 15.5	0.646
Height, cm	170.8 ± 8.0	171.3 ± 5.5	0.894	171.4 ± 12.4	170.5 ± 6.8	0.851
BMI,	25.2 ± 2.0	27.9 ± 3.6	0.061	24.2 ± 4.0	23.2 ± 4.1	0.602
*History of disease*						
HTN	8 (80.0)	10 (100.0)	0.279	0 (0.0)	0 (0.0)	N.A
Diabetes	4 (40.0)	0 (0.0)	0.033	1 (10.0)	0 (0.0)	0.305
Hepatitis	1 (10.0)	0 (0.0)	0.330	1 (10.0)	0 (0.0)	0.305
Tuberculosis	0 (0.0)	0 (0.0)	N.A	0 (0.0)	0 (0.0)	N.A
*Blood tests*						
ALT, U/L	14.0 (8.0–16.0)	15.0 (12.0–20.0)	0.293	16.0 (13.0–21.0)	17.5 (12.0–52.0)	0.483
AST, U/L	14.5 (13.0–22.0)	16.0 (15.0–18.0)	0.493	14.0 (13.0–28.0)	24.5 (15.0–52.0)	0.087
Creatine, mg/dL	268.0 (129.0–337.0)	160.0 (136.0–230.0)	0.094	156.5 (124.0–242.0)	82.5 (64.0–91.0)	0.002
Urea, mg/dL	16.5 (15.9–22.7)	8.4 (5.5–9.1)	0.006	11.1 (6.8–16.2)	5.2 (3.9–6.6)	0.022
eGFR, ml/min/l.73 m^2^	36.6 (15.0–54.0)	47.1 (32.1–53.9)	0.204	50.8 (24.8–60.0)	99.4 (71.5–117.2)	0.003
24-h urinary protein, mg/24 h	0.9 (0.2–1.7)	0.5 (0.3–1.3)	0.541	0.5 (0.1–2.3)	2.5 (0.6–8.8)	0.162
HbA1c, %	6.0 (5.3–7.1)	5.4 (5.2–5.8)	0.111	5.1 (4.8–5.8)	4.9 (4.8–6.8)	0.352
Fasting Glucose, mmol/L	8.1 (6.4–11.6)	6.2 (6.0–9.1)	0.322	5.3 (4.9–7.9)	5.5 (5.1–8.5)	0.813
HDL cholesterol, mmol/L	0.8 (0.7–0.8)	0.9 (0.8–1.0)	0.263	0.9 (0.8–1.2)	1.6 (1.5–1.7)	0.009
LDL cholesterol, mmol/L	2.4 (1.3–3.3)	2.8 (2.2–3.0)	0.684	2.4 (1.9–7.3)	4.2 (2.0–6.0)	0.413
Triglycerides (mg/dL)	2.0 (1.4–4.3)	1.7 (1.6–2.4)	0.205	1.7 (1.3–2.9)	1.8 (1.4–2.3)	0.579
Total cholesterol, mmol/L	4.3 (3.9–6.2)	4.3 (3.9–4.8)	0.366	4.4 (3.3–9.8)	6.7 (4.1–8.0)	0.379
*Health-related behavior*						
Smoking status	3 (30.0)	1 (10.0)	0.313	2 (20.0)	2 (20.0)	1.000
Alcohol drinking	3 (30.0)	0 (0.0)	0.073	1 (10.0)	0 (0.0)	0.305
*Discharge medications*						
Aspirin	0 (0.0)	2 (20.0)	0.456	0 (0.0)	0 (0.0)	N.A
Beta-blocker	1 (10.0)	1 (10.0)	1.000	0 (0.0)	0 (0.0)	N.A
Diuretics	0 (0.0)	0 (0.0)	N.A	0 (0.0)	1 (10.0)	1.000
Lipid-lowering drugs	0 (0.0)	0 (0.0)	N.A	0 (0.0)	1 (10.0)	1.000
Calcium channel blockers	3 (30.0)	0 (0.0)	0.210	1 (10.0)	0 (0.0)	1.00
Antihypertensive drugs	5 (50.0)	3 (30.0)	0.648	0 (0.0)	0 (0.0)	N.A
Xanthine oxidase inhibitor	2 (20.0)	0 (0.0)	0.456	1 (10.0)	0 (0.0)	1.000

ALT: alanine aminotransferase; AST: aspartate aminotransferase; BMI: body mass index; BP: blood pressure; eGFR: estimated glomerular filtration rate; sUA: serum uric acid; HDL: high-density lipoprotein; HTN: hypertension; LDL: low-density lipoprotein; Data are presented as No. (%), mean ± SD or median (IQR); *p values were calculated by using T test or Wilcoxon test for continuous variables and *χ*^2^ test or fisher exact test for categorical variable.

## Discussion

Our investigation has corroborated the presence of an additive interactive effect between serum uric acid (sUA) levels and hypertension on the risk of chronic kidney disease (CKD), with a stronger association observed in the upper fourth quartile of sUA among hypertensive subjects compared to non-hypertensive individuals. The nuance of sUA control, especially in the context of hypertension, cannot be overstated.

The CKD incidence rates in our study—10.2% for hypertensive subjects and 3.9% for non-hypertensive subjects—are consistent with previous findings within China [25]. The direct impact of hypertension and sUA on CKD development has been substantiated by several epidemiological and mechanistic studies [26–28]. A pronounced interaction between hypertension and sUA quartiles on CKD was evident, with the highest CKD risk identified in hypertensive patients, particularly those with elevated systolic blood pressure and in the highest sUA quartile. These findings contrast with earlier research indicating a decline in CKD prevalence solely among hypertensive subjects managed with antihypertensive medications, including renin-angiotensin-aldosterone system (RAS) inhibitors and calcium channel blockers (CCBs) [29]. Our analysis did not account for RAS inhibitor and CCB usage, yet it revealed a substantial risk of CKD with concurrent high sUA and hypertension. Despite this correlation, mediation analysis suggested hypertension may not serve as a mediating factor in sUA-induced CKD [30].

Further investigation into the mechanistic role of sUA and hypertension in CKD risk involved renal biopsy analyses from Zhongshan Hospital. Elevated numbers of CD4+ T cells and macrophages in hypertensive subjects with increased sUA levels suggest a ‘double hit’ model of uric acid amplifying hypertension-induced renal damage and contributing to tubulointerstitial inflammation. Such inflammatory environments can exacerbate systemic hypertension and CKD progression. Previous studies also identified increased sUA levels as stimulators of the RAS, leading to endothelial damage and oxidative stress at both cellular and tissue levels [31,32], potentially precipitating vascular and tubulointerstitial lesions that expedite CKD progression [14]. However, urine albumin value was not collected from CHARLS. Albuminuria is a sign of established organ damage; GFR is mostly a measure of the dynamic function of the kidneys [33]. In a cross-sectional study among a sub-Saharan African population, elevated sUA levels were significantly associated with kidney microvascular dysfunction (albuminuria) and mediated partly through elevated blood pressure [34]. On the contrary, Elisa Russo. et al. reported that there was an inverse relationship between sUA and the presence of macroalbuminuria after adjustment for potential confounding factors [35]. Future studies need to clarify the relationship among sUA values, eGFR, and albuminuria, which may shed light on the mechanisms underlying the excess of renal injury associated with both sUA and hypertension and lead to better risk stratification.

The relationship between sUA levels and CKD remains a subject of debate, with some studies suggesting a nonlinear increase in CKD risk at higher sUA concentrations [36], while others report a consistent trend of elevated sUA as an independent risk factor for reduced kidney function [13,37]. Such discrepancies may stem from the heterogeneity of study populations and whether elevated sUA levels are primary (e.g. dietary) or secondary (e.g. due to reduced renal excretion or metabolic disease) [38]. It could also be explained that it may relate to whether there is crystal deposition in the kidney, which might be expected to be higher among individuals with gout, although people with asymptomatic hyperuricemia may also have silent crystal deposition in joints and other organs [39,40].

Our study also found that the CKD risk was significantly increased with the rising sUA quartiles. There existed no evidence for a non-linear J or U-shaped relationship between sUA and CKD outcomes. The relationship between sUA and CKD in the hypertensive and non-hypertensive groups confirmed that a high level of sUA represents a high-risk state for CKD. In the study, the risk ratios for CKD were initiated and strengthened with sUA levels of ≥ 4.55 mg/dL in the hypertensive population and ≥ 5.58 mg/dL in the non-hypertensive population. As a lower value of sUA/sCr is probably a measure of lesser production of uric acid or a greater capacity of the body to excrete it through the kidney or the gut, we analyzed sUA/sCr and CKD [41]. In agreement with the study of Nathalia Rabello Silva et al. when we analyzed the sUA/sCr, there was a negative association with CKD among hypertensive individuals [42]. The data might be explained by that individuals with hypertension are often accompanied by renal excretion dysfunction. This could also explain the low threshold in the hypertensive population in our study.

These findings suggested that the risk effect of low-medium sUA levels on CKD in hypertensive patients might be strengthened compared with those in non-hypertensive individuals. The distinct threshold range of sUA and CKD among hypertensive vs. non-hypertensive individuals is intriguing in light of previous findings, suggesting that target urate-lowering therapy (ULT) may be initiated toward different sUA values based on accompanied disease states. Given the disparity of sUA on CKD risk among hypertensive vs. non-hypertensive individuals, the question should not be if, but rather where, patients with different sUA threshold ranges should be treated with the ULT. Therefore, it is important to differentiate between sUA control in ① pre-hypertension, ② early hypertension, and ③ late hypertension with complications.

The major strengths of this study are the large sample size from a well-established cohort in China, which allowed us to perform joint and stratified analyses with sufficient statistical power. Several limitations should be noted. First, the time-varying changes in sUA were not collected in this study. The single-time detection of the established design in the CHARLS limited the time-varying changes. A new detection method is needed to assess the dynamic threshold of sUA with CKD development. Second, owing to the nature of subgroup analyses, the sample size in each subgroup was not calculated before data collection. Especially, the number of participants and events might be insufficient among sUA Q1 subgroups in hypertensive patients and the results should be cautiously interpreted. Finally, although our study controlled for key individuals’ characteristics and comorbidities, residual confounding was still possible.

## Conclusions

Drawing from a robust nationwide cohort, our findings underscore the pivotal role of hypertension and elevated sUA in fostering CKD development. Importantly, individuals with concurrent hypertension and high sUA levels face an increased CKD risk. These insights underscore the need for vigilant monitoring and management of moderate to high sUA levels in hypertensive patients in China. This research contributes significantly to ongoing efforts to prevent CKD, particularly in relation to hypertension and sUA.

## Supplementary Material

Supplemental Material

## Data Availability

The data underlying this article are available in CHARLS Database at http://charls.pku.edu.cn/.
