# Supplementary material for: Disparity of serum uric acid threshold for CKD among hypertensive and non-hypertensive individuals
Source: Ren Fail. 2024 Feb 29;46(1):2301041. doi: 10.1080/0886022X.2023.2301041 (PMC10911134; doi:10.1080/0886022X.2023.2301041)
Supplement: Supplemental Material [file IRNF_A_2301041_SM2013.pdf]

Supplementary Material

Bowen Zhu, Fang Li, Weidong Zhang, Nana Song, ShuanZhao, Shi Jin, Yufei Lu, Liwen Zhang, Yang Li, Hong Liu

Supplementary Table 1 Multivariate predictors of sUA for Chronic Kidney Disease in population with and without elevated Systolic BP

Supplementary Table 2 Multivariate predictors of sUA for Chronic Kidney Disease in population with and without elevated Diastolic BP

Supplementary Table 3 Risk of Chronic Kidney Disease by threshold sUA according to hypertension status in all population and propensity score matching population

Supplementary Figure 1 Flow diagram for selecting participants

Supplementary Figure 2 Flow diagram for selecting participants in the nephrology department of Zhongshan Hospital

Information Classification: General

Supplementary Table 1 Multivariate predictors of sUA for Chronic Kidney Disease in population with and without elevated Systolic BP

	Incidence of CKD [n (%)]	Model 1		Model 2	
		Risk ratio (95% confidence interval)	*P-value*	Risk ratio (95% confidence interval)	*P-value*
Elevated Systolic BP (n = 1639)					
Q1 (Male: <4.2; Female: <3.7)	11 (3.5)	1.00 (ref)		1.00 (ref)	
Q2 (Male: 4.2–4.9; Female: 3.7–3.9)	38 (10.0)	2.2 (1.1–4.7)	0.037	1.9 (0.9–4.0)	0.11
Q3 (Male: 4.9–6.4; Female:4.0–5.3)	81 (16.6)	3.0 (1.4–6.2)	0.004	2.7 (1.3–5.7)	0.009
Q4 (Male:> 6.4; Female: >5.3)	185 (31.2)	7.5 (3.8–14.7)	<.001	7.1 (3.6–14.1)	<.001
Normal Systolic BP (n = 2541)					
Q1 (Male: <4.2; Female: <3.7)	21 (2.2)	1.00 (ref)		1.00 (ref)	
Q2 (Male: 4.2–4.9; Female: 3.7–3.9)	40 (4.4)	1.9 (0.8–4.6)	0.154	1.7 (0.7–4.1)	0.248
Q3 (Male: 4.9–6.4; Female:4.0–5.3)	61 (7.9)	4.1 (1.9–9.2)	<.001	3.7 (1.6–8.3)	0.002
Q4 (Male:> 6.4; Female: >5.3)	101 (14.7)	7.2 (3.3–15.8)	<.001	6.3 (2.8–13.8)	<.001
Indices of "hypertension+sUA" : RERI: 1.122 95% CI (0.372–1.873); AP: 0.264 95% CI(0.193–0.335); SI: 1.528 95%CI (1.314–1.778).					
Propensity Elevated Systolic BP (n = 1380)					
Q1 (Male: <4.2; Female: <3.7)	7 (2.1)	1.00 (ref)		1.00 (ref)	

Information Classification: General

Q2 (Male: 4.2–4.9; Female: 3.7–3.9)	18 (5.5)	2.4 (1.0–6.0)	0.054	2.4 (1.0–5.9)	0.063
Q3 (Male: 5.0–6.3; Female:4.0–5.3)	27 (8.0)	4.0 (1.7–9.4)	0.002	4.1 (1.7–9.8)	0.001
Q4 (Male:> 6.3; Female: >5.3)	58 (15.1)	7.8 (3.5–17.6)	<.001	8.1 (3.6–18.5)	<.001
Matched Normal Systolic BP (n = 1334)					
Q1 (Male: <4.2; Female: <3.7)	6 (1.7)	1.00 (ref)		1.00 (ref)	
Q2 (Male: 4.2–4.9; Female: 3.7–3.9)	9 (2.6)	1.4 (0.5–4.0)	0.542	1.3 (0.5–3.9)	0.586
Q3 (Male: 5.0–6.3; Female:4.0–5.3)	20 (5.9)	3.5 (1.4–9.1)	0.008	3.4 (1.3–8.7)	0.012
Q4 (Male:> 6.3; Female: >5.3)	31 (10.5)	6.9 (2.8–17.1)	<.001	6.5 (2.6–16.3)	<.001

Indices of "hypertension+sUA" : RERI: 0.525 95% CI (-0.041–1.094); AP: 0.167 95% CI (0.064–0.271); SI: 1.325 95%CI (1.099–1.598).

Abbreviation: sUA, serum uric acid. Model 1: age and gender adjustment; Model 2: age, gender, BMI, education, residence, waist circumance, education, diabetes, drinking, smoking and anti-hypertensive drugs adjustment.

Supplementary Table 2 Multivariate predictors of sUA for Chronic Kidney Disease in population with and without elevated Diastolic BP

	Incidence of CKD [n (%)]	Model 1		Model 2	
		Risk ratio (95% confidence interval)	*P-value*	Risk ratio (95% confidence interval)	*P-value*
Elevated Diastolic BP (n = 1639)					
Q1 (Male: <4.2; Female: <3.7)	1 (0.6)	1.00 (ref)		1.00 (ref)	

Information Classification: General

Q2 (Male: 4.2–4.9; Female: 3.7–3.9)	6 (3.1)	4.1 (0.5–34.7)	0.199	3.7 (0.4–32.1)	0.231
Q3 (Male: 4.9–6.4; Female:4.0–5.3)	8 (3.5)	5.1 (0.6–42.0)	0.128	4.0 (0.5–33.7)	0.201
Q4 (Male:> 6.4; Female: >5.3)	47 (16.7)	30.7 (4.1–227.1)	<.001	28.9 (3.9–215.1)	0.001
Normal Diastolic BP (n = 2541)					
Q1 (Male: <4.2; Female: <3.7)	17 (2.0)	1.00 (ref)		1.00 (ref)	
Q2 (Male: 4.2–4.9; Female: 3.7–3.9)	36 (4.2)	2.0 (1.1–3.7)	0.02	1.8 (1.0–3.3)	0.065
Q3 (Male: 4.9–6.4; Female:4.0–5.3)	55 (6.7)	3.5 (2.0–6.2)	<.001	3.2 (1.8–5.7)	<.001
Q4 (Male:> 6.4; Female: >5.3)	95 (12.5)	5.9 (3.4–10.1)	<.001	5.3 (3.1–9.3)	<.001
Indices of "hypertension+sUA" : RERI: -0.279 95% CI (-0.605–0.048); AP: -0.321 95% CI (-0.900–0.257); SI: -0.910 95%CI (-0.944–0.386).					
Elevated Diastolic BP (n = 709)					
Q1 (Male: <4.2; Female: <3.7)	0 (0.0)	1.00 (ref)		1.00 (ref)	
Q2 (Male: 4.2–4.9; Female: 3.7–3.9)	5 (3.3)	N.A		N.A	
Q3 (Male: 5.0–6.3; Female:4.0–5.3)	6 (3.3)	N.A		N.A	
Q4 (Male:> 6.3; Female: >5.3)	25 (12.3)	N.A		N.A	
Matched normal Diastolic BP (n = 2005)					
Q1 (Male: <4.2; Female: <3.7)	13 (2.6)	1.00 (ref)		1.00 (ref)	

Information Classification: General

Q2 (Male: 4.2–4.9; Female: 3.7–3.9)	22 (4.2)	1.6 (0.8–3.2)	0.200	1.6 (0.8–3.3)	0.194
Q3 (Male: 5.0–6.3; Female:4.0–5.3)	41 (8.3)	3.4 (1.8–6.6)	<.001	3.4 (1.8–6.6)	<.001
Q4 (Male:> 6.3; Female: >5.3)	64 (13.5)	5.7 (3.0–10.6)	<.001	5.6 (2.9–10.5)	<.001
Indices of "hypertension+sUA" : RERI: -0.211 95% CI (-0.532–0.110); AP: -0.278 95% CI (-0.886–0.330); SI: N.A.					

Abbreviation: sUA, serum uric acid. Model 1: age and gender adjustment; Model 2: age, gender, BMI, education, residence, waist circumance, education, diabetes, drinking, smoking and anti-hypertensive drugs adjustment.

Information Classification: General

Supplementary Table 3 Risk of Chronic Kidney Disease by threshold sUA according to hypertension status in all population and propensity score matching population

Threshold SUA, mg/dL	All population		PSM population	
	Multivariable adjusted RR (95% CI)		Multivariable adjusted RR (95% CI)	
	Hypertension	Non-hypertension	Hypertension	Non-hypertension
3.0	3.91 (1.20–12.73)*	2.94 (1.05–8.25)*	4.73 (1.12–20.06)*	4.05 (0.95–17.18)
3.1	3.66 (1.31–10.27)*	2.94 (1.16–7.47)*	3.92 (1.19–12.94)*	3.27 (0.99–10.82)
3.2	3.25 (1.38–7.67)**	2.94 (1.25–6.93)**	3.73 (1.31–10.63)*	3.91 (1.19–12.90)*
3.3	4.02 (1.72–9.42)***	2.40 (1.17–4.95)***	4.50 (1.59–12.76)*	2.96 (1.14–7.67)*
3.4	4.09 (1.85–9.05)***	2.47 (1.23–4.93)***	4.24 (1.65–10.88)*	3.41 (1.32–8.83)*
3.5	4.96 (2.25–10.93)***	2.29 (1.22–4.27)***	5.02 (1.96–12.83)*	2.91 (1.28–6.63)**
3.6	5.76 (2.74–12.11)***	2.63 (1.43–4.85)***	5.53 (2.33–13.08)**	3.20 (1.46–7.03)***
3.7	5.28 (2.69–10.34)***	2.66 (1.49–4.75)***	5.50 (2.46–12.27)**	2.87 (1.40–5.90)***
3.8	5.35 (2.87–9.99)***	2.86 (1.62–5.06)***	5.80 (2.71–12.00)**	2.99 (1.49–6.00)***
3.9	4.94 (2.80–8.74)***	2.93 (1.70–5.06)***	5.64 (2.81–11.30)**	2.85 (1.48–5.49)***
4.0	4.70 (2.79–7.90)***	3.20 (1.87–5.48)***	4.72 (2.56–8.70)**	3.00 (1.57–5.70)***
4.1	4.42 (2.72–7.20)***	3.40 (2.00–5.78)***	4.05 (2.31–7.10)**	3.34 (1.76–6.34)***
4.2	4.60(2.86–7.37)***	3.12 (1.89–5.15)***	4.36 (2.52–7.56)***	3.22 (1.75–5.91)***
4.3	4.46 (2.85–6.98)***	2.94 (1.81–4.77)***	4.25 (2.52–7.18)***	2.93 (1.64–5.22)***
4.4	4.27 (2.78–6.57)***	3.16 (1.96–5.10)***	4.34 (2.60–7.24)***	3.01 (1.71–5.29)***
4.5	3.86 (2.56–5.82)***	3.49 (2.17–5.61)***	3.70 (2.28–6.00)***	3.26 (1.87–5.71)***
4.6	3.55 (2.39–5.28)***	3.10 (1.94–4.95)***	3.47 (2.17–5.56)***	3.10 (1.78–5.38)***
4.7	3.37 (2.30–4.95)***	3.04 (1.91–4.85)***	3.20 (2.03–5.06)***	3.08 (1.77–5.33)***
4.8	3.49 (2.38–5.09)***	3.26 (2.05–5.20)***	3.61 (2.29–5.69)***	3.25 (1.88–5.63)***
4.9	3.47 (2.38–5.05)***	3.36 (2.11–5.35)***	3.40 (2.16–5.34)***	3.39 (1.96–5.87)***
5.0	3.30 (2.27–4.80)***	3.18 (1.99–5.07)***	3.34 (2.12–5.25)***	3.23 (1.87–5.60)***

Information Classification: General

5.1	3.19 (2.19–4.63)***	3.11 (1.95–4.96)***	3.33 (2.11–5.24)***	3.43 (1.99–5.93)***
5.2	2.92 (2.01–4.25)***	3.10 (1.92–5.00)***	2.96 (1.88–4.60)***	3.23 (1.85–5.63)***
5.3	3.04 (2.08–4.44)***	3.43 (2.10–5.61)***	3.28 (2.06–5.22)***	3.75 (2.12–6.63)***
5.4	3.26 (2.22–4.78)***	3.37 (2.04–5.56)***	3.43 (2.15–5.46)***	4.06 (2.28–7.23)***
5.5	3.17 (2.16–4.66)***	3.56 (2.16–5.88)***	3.26 (2.04–5.19)***	4.27 (2.40–7.60)***
5.6	3.27 (2.21–4.84)***	3.20 (1.91–5.35)***	3.40 (2.12–5.47)***	3.60 (1.99–6.49)***
5.7	3.22 (2.17–4.77)***	2.79 (1.63–4.77)***	3.39 (2.10–5.47)***	2.74 (1.49–5.04)***
5.8	3.04 (2.03–4.53)***	3.00 (1.72–5.21)***	3.44 (2.13–5.57)***	2.88 (1.52–5.45)***
5.9	3.15 (2.09–4.75)***	2.51 (1.40–4.50)***	3.51 (2.15–5.72)***	2.18 (1.11–4.31)***
6.0	3.08 (2.02–4.69)***	2.66 (1.47–4.83)***	3.61 (2.19–5.95)***	2.53 (1.28–5.04)***
6.1	3.22 (2.08–4.98)***	2.83 (1.51–5.29)***	3.85 (2.31–6.43)***	2.52 (1.21–5.23)***
6.2	3.53 (2.25–5.53)***	3.34 (1.78–6.26)***	4.05 (2.39–6.88)***	2.98 (1.43–6.20)***
6.3	3.48 (2.18–5.56)***	3.06 (1.58–5.94)***	4.18 (2.40–7.29)***	2.55 (1.16–5.60)***
6.4	3.65 (2.27–5.88)***	3.55 (1.82–6.93)***	4.74 (2.70–8.32)***	2.99 (1.36–6.60)***
6.5	3.68 (2.25–6.02)***	3.19 (1.57–6.47)***	4.74 (2.64–8.49)***	3.43 (1.54–7.64)***
6.6	3.96 (2.39–6.56)***	2.66 (1.22–5.80)***	5.10 (2.80–9.30)***	3.22 (1.37–7.58)***
6.7	4.07 (2.44–6.79)***	2.86 (1.25–6.50)***	5.28 (2.87–9.74)***	3.44 (1.39–8.52)***
6.8	3.57 (2.09–6.11)***	3.19 (1.39–7.34)***	3.95 (2.08–7.52)***	3.81 (1.52–9.54)***
6.9	3.60 (2.02–6.40)***	3.70 (1.58–8.65)***	4.22 (2.13–8.37)***	4.69 (1.82–12.07)**
7.0	3.47 (1.91–6.33)***	3.72 (1.52–9.14)***	4.35 (2.13–8.86)***	4.83 (1.76–13.24)**
7.1	3.60 (1.94–6.68)***	4.2 (1.70–10.37)***	4.43 (2.12–9.29)***	5.39 (1.96–14.83)**
7.2	4.60 (2.43–8.69)***	3.32 (1.19–9.30)***	5.24 (2.46–11.13)***	3.77 (1.18–12.05)**
7.3	4.80 (2.36–9.76)***	4.74 (1.71–13.15)**	5.66 (2.47–12.98)***	4.92 (1.51–15.99)**
7.4	5.53 (2.62–11.68)***	5.73 (2.02–16.26)**	8.27 (3.43–19.97)***	6.00 (1.79–20.00)**
7.5	6.09 (2.80–13.25)***	5.18 (1.63–16.45)*	9.13 (3.65–22.79)**	4.95 (1.26–19.48)**
7.6	8.07 (3.52–18.50)***	6.88 (2.16–21.93)**	14.98 (5.40–41.6)**	6.27 (1.59–24.68)**

Information Classification: General

7.7	8.58 (3.52–20.94)*	7.63 (2.36–24.69)**	11.88 (4.04–34.94)**	6.98 (1.74–28.08)
7.8	10.28 (3.99–26.51)	7.86 (2.09–29.61)*	16.83 (5.17–54.74)*	6.40 (1.25–32.70)*
7.9	14.53 (5.09–41.48)	10.19 (2.66–39.00)*	18.05 (5.02–64.88)*	7.71 (1.47–40.41)*
8.0	14.78 (5.14–42.48)	11.09 (2.86–43.04)*	18.44 (5.08–66.95)**	8.46 (1.58–45.31)*

Abbreviation: sUA, serum uric acid; RR, risk ratio. RR was adjusted for age, gender, BMI, education, residence, waist circumstance, education, diabetes, drinking, smoking and anti-hypertensive drugs. * P<0.05; ** P<0.01; *** P<0.001.

Information Classification: General

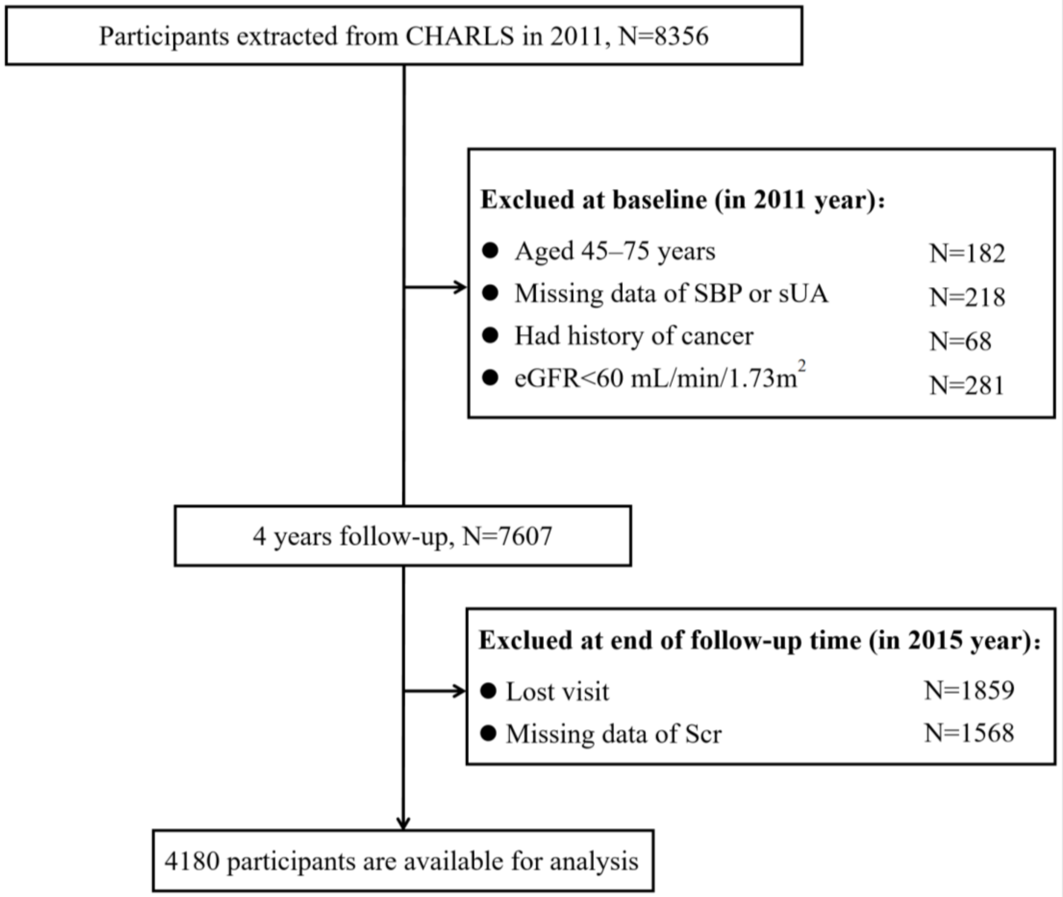

Supplementary Figure 1 Flow diagram for selecting participants.

Information Classification: General

Supplementary Figure 2 Flow diagram for selecting participants in the nephrology department of Zhongshan Hospital.

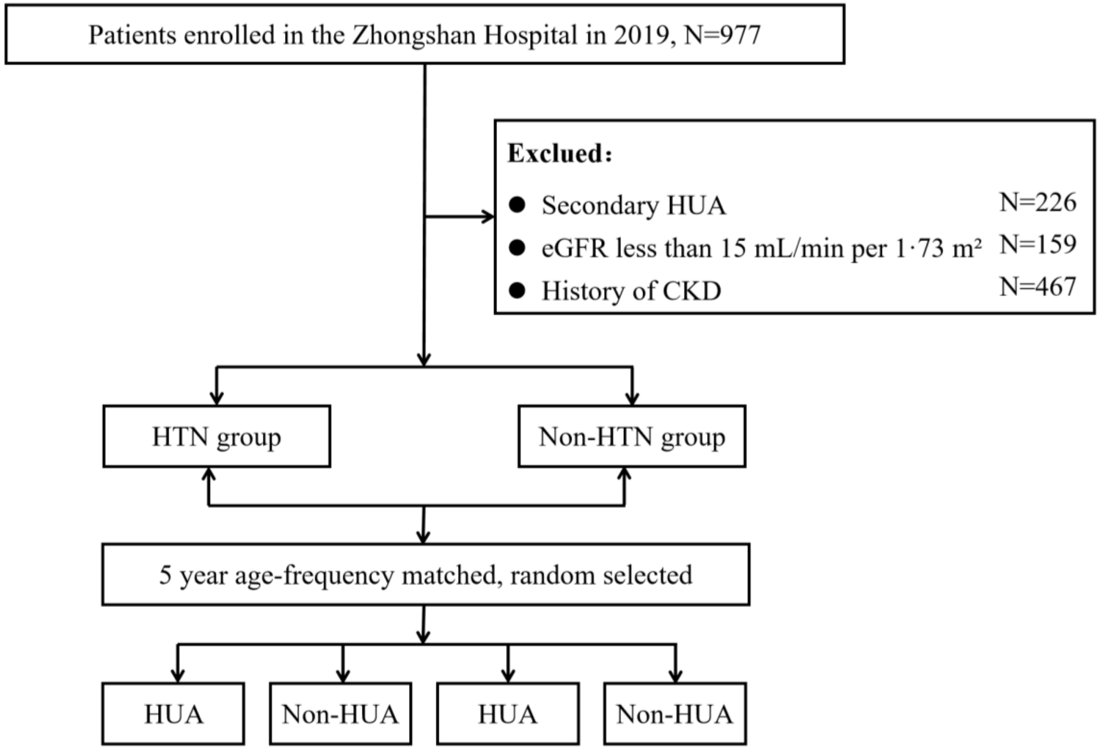

Information Classification: General
